# Supplementary material for: What Can We Learn about the Bias of Microbiome Studies from Analyzing Data from Mock Communities?
Source: Genes (Basel). 2022 Sep 28;13(10):1758. doi: 10.3390/genes13101758 (PMC9601962; doi:10.3390/genes13101758)
Supplement: Supplementary file 1 [file genes-13-01758-s001.zip › genes-1889232-supplementary.pdf]

Supplementary Material

Supplemental Text S1: DNA Extraction Efficiency

The amount of DNA extracted from each sample could possibly be related to extraction efficiency. Thus, any systematic differences in the quantity of DNA extracted from samples when using different protocols might explain differences in the observed biases, although this effect would indicate a violation of the MWC bias model. Although Zhao and Satten found certain departures from the MWC approach were small if they existed at all, we nonetheless examined whether there was an association between protocol and the amount of DNA extracted. Using ANOVA we found that, while samples preserved in Lifeguard® produced on average slightly higher amounts of DNA than the other protocols (Lab supplement Figure 1), these effects were not significant (p=0.612). Further, we did not note any consistent effect of extraction protocol on DNA concentrations when looking across different product types.

Significant differences were observed using ANOVA (p < 0.0001) in product-to-product extraction amounts, where Grizzly LC Premium Natural had the highest amount of DNA in extracts at 15.7 ng/ul, and Longhorn LC Natural tended to have the lowest DNA extracted at 3.32 ng/ul. These ANOVA calculations were performed using JMP in SAS.

Figure S1: DNA concentration by extraction protocol.

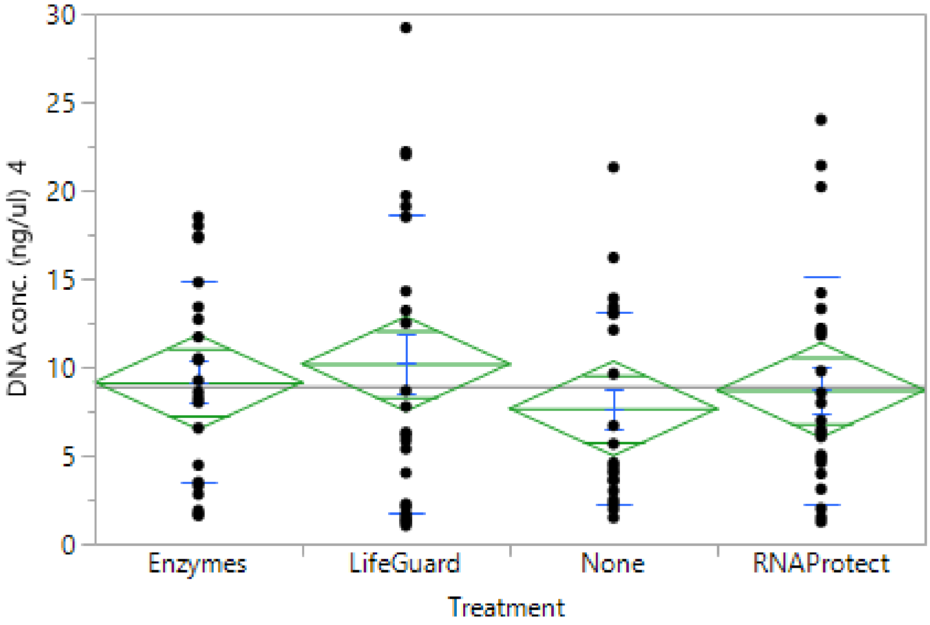

Figure S2: DNA concentration by product.

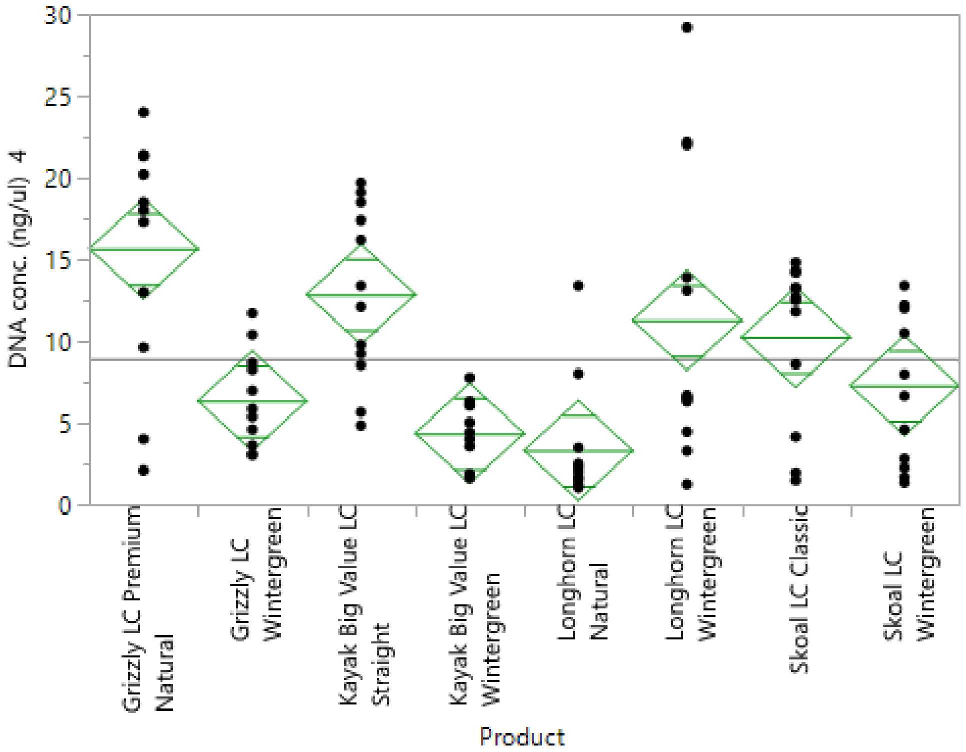

Analysis Results for ZMC and ZMC+Matrix Excluding *Pseudomonas*

Table S1. Log-Bias Factors for taxa in ZMC and ZMC+Matrix samples.

Taxon	Base	Enzymes	Lifeguard	RNAProtect
ZMC				
Bacillus	0.697	0.485	0.699	0.847
Listeria	0.302	0.488	0.331	0.261
Staphylococcus	-0.054	0.090	0.090	-0.225
Enterococcus	0.035	-0.096	0.088	0.033
Lactobacillus	-0.071	0.007	0.047	-0.246
Escherichia/Shigella	-0.447	-0.478	-0.624	-0.327
Salmonella	-0.461	-0.496	-0.631	-0.343
Effect Size	0.143	0.138	0.200	0.160
p-value	0.0007	0.0024	0.0003	$<1 \times 10^{-4}$
ZMC+Matrix				
Bacillus	0.660	-0.007	0.773	0.720
Listeria	0.417	0.375	0.379	0.304
Staphylococcus	-0.352	0.295	-0.284	-0.753
Enterococcus	0.344	-0.428	0.212	0.004
Lactobacillus	0.368	0.382	0.271	0.238
Escherichia/Shigella	-0.702	-0.305	-0.659	-0.248
Salmonella	-0.735	-0.312	-0.690	-0.264
Effect Size	0.289	0.107	0.264	0.195
p-value	$<1 \times 10^{-4}$	0.0002	$<1 \times 10^{-4}$	$<1 \times 10^{-4}$

Table S2. Effect sizes and *p*-values for tests of pairwise bias differences for four extraction protocols in ZMC (lower triangle) and Zymo+Matrix (upper triangle) samples. Calculated without *Pseudomonas*.

ZMC+Matrix (Overall: 0.159 ($p \ll 1 \times 10^{-4}$))

ZMC (Overall: 0.030 (p=0.1280))

	Base	Enzymes	Lifeguard	RNAProtect
Base		0.257 (p<1e-04)	0.007 (p=0.3506)	0.105 (p=0.0002)
Enzymes	0.018 (p=0.3527)		0.234 ($p \ll 1 \times 10^{-4}$)	0.264 ($p \ll 1 \times 10^{-4}$)
Lifeguard	0.014 (p=0.4453)	0.021 (p=0.2828)		0.089 (p=0.0019)
RNAProtect	0.016 (p=0.4067)	0.058 (p=0.0591)	0.055 (p=0.0879)	

Table S3. Tests of effect of Snus Matrix on bias parameters for four extraction protocols, calculated without *Pseudomonas*.

	p-value (10,000 permutation replicates)				
	Base	Enzymes	Lifeguard	RNAProtect	Overall
Effect Size	0.076	0.087	0.031	0.078	0.068
p-value	0.0086	0.0084	0.2104	0.0050	0.0012

Table S4. Scaled pairwise Euclidean distances (divided by their Frobenius norms) between four extraction protocols for ZMC, ZMC+Matrix and tobacco samples. Calculated without *Pseudomonas*

Protocol pairs	ZMC	ZMC+Matrix	tobacco
Base vs. Enzymes	0.2219	0.3667	0.3564
Base vs. Lifeguard	0.1964	0.0609	0.1776
Base vs. RNAProtect	0.2098	0.2349	0.1830
Enzymes vs. Lifeguard	0.2387	0.3495	0.3769
Enzymes vs. RNAProtect	0.4000	0.3714	0.3700
Lifeguard vs. RNAProtect	0.3888	0.2159	0.1704

Table S5. Mean-centered log-bias factors for four extraction methods in 10 taxa in six smokeless tobacco products.

Taxon	Base	Enzymes	Lifeguard	RNAProtect
Corynebacterium	-0.005	-0.049	-0.016	0.069
Corynebacteriaceae	-0.043	-0.003	0.014	0.032
Brevibacterium	-0.052	-0.098	0.069	0.080
Bacillus	-0.038	0.373	-0.154	-0.181
Staphylococcus	-0.059	0.040	0.128	-0.109
Atopostipes	-0.104	-0.073	-0.043	0.074
Marinilactibacillus	0.089	-0.163	0.080	-0.005
Tetragenococcus	0.032	-0.052	0.010	0.011
Lactobacillus	0.015	0.088	-0.109	0.006
Enteractinococcus	0.166	-0.208	0.021	0.022
